# Geographical patterns of social cohesion drive disparities in early COVID infection hazard

**DOI:** 10.1073/pnas.2121675119

**Published:** 2022-03-14

**Authors:** Loring J. Thomas, Peng Huang, Fan Yin, Junlan Xu, Zack W. Almquist, John R. Hipp, Carter T. Butts

**Affiliations:** ^a^Department of Sociology, University of California, Irvine, CA 92697;; ^b^Department of Statistics, University of California, Irvine, CA 92697;; ^c^Department of Sociology, University of Washington, Seattle, WA 98195;; ^d^Department of Statistics, University of Washington, Seattle, WA 98195;; ^e^Center for Studies in Demography and Ecology, University of Washington, Seattle, WA 98195;; ^f^Center for Statistics and the Social Sciences, University of Washington, Seattle, WA 98195;; ^g^eScience Institute, University of Washington, Seattle, WA 98195;; ^h^Department of Criminology, Law & Society, University of California, Irvine, CA 92697;; ^i^Department of Computer Science, University of California, Irvine, CA 92697;; ^j^Department of Electrical Engineering and Computer Science, University of California, Irvine, CA 92697

**Keywords:** COVID-19, spatial heterogeneity, diffusion, health disparities, social networks

## Abstract

The uneven spread of COVID-19 has resulted in disparate experiences for marginalized populations in urban centers. Using computational models, we examine the effects of local cohesion on COVID-19 spread in social contact networks for the city of San Francisco, finding that more early COVID-19 infections occur in areas with strong local cohesion. This spatially correlated process tends to affect Black and Hispanic communities more than their non-Hispanic White counterparts. Local social cohesion thus acts as a potential source of hidden risk for COVID-19 infection.

The spread of COVID-19 has infected millions globally (1) and, in the United States, this has disproportionately affected Black and Latino populations (2). The COVID-19 pandemic has been shown to spread unevenly over social and geographic space (3–5); however, the mechanistic connections between contact network structure and infection hazard are not fully understood. Here, we show that small differences in local social cohesion can result in large disparities in infection rates by race and ethnicity as observed in the United States (6).

While long-term outcomes are important, we specifically aim to understand how the disparities in infection by race and ethnicity arise early in the pandemic. In the initial phase of an emerging pandemic, risks are unclear, nonpharmaceutical interventions (e.g., masking, distancing) are not yet implemented, and behavioral changes are rarely widespread; yet it is precisely at this point that the virus has the greatest opportunity to penetrate the population, with the capacity to provide particular harms to vulnerable communities.

Using a previously published explicit contact network model based on viral dynamics in the early COVID-19 pandemic (3), we examine the network properties that drive differences in initial infection hazard. As Fig. 1 shows, wild-type severe acute respiratory syndrome coronavirus 2 (SARS-CoV-2) does not diffuse readily through linear “infection chains” with multiple intermediates; even when multiple, parallel chains connect two individuals, many chains are required to achieve a large infection risk. By contrast, SARS-CoV-2 spreads extremely well through cohesive subgroups, where multiple, redundant ties provide numerous avenues for infection to occur. Being connected to an infective by shared membership in even a fairly small cohesive group results in a dramatic increase in infection risk, due to the factorial increase in the number of potential infection paths with group size. For example, an otherwise isolated susceptible linked to an infective via a clique of only six individuals has a 50% probability of becoming infected; to reach the same infection probability by connection with independent paths of the type shown in Fig. 1 would require maintaining 38 contacts involving 76 intermediaries. This suggests that small differences in social cohesion can lead to large disparities in infection risk for wild-type SARS-CoV-2, much as small differences in partnership concurrency have been shown to drive disparities in HIV risk (7).

**Fig. 1.**
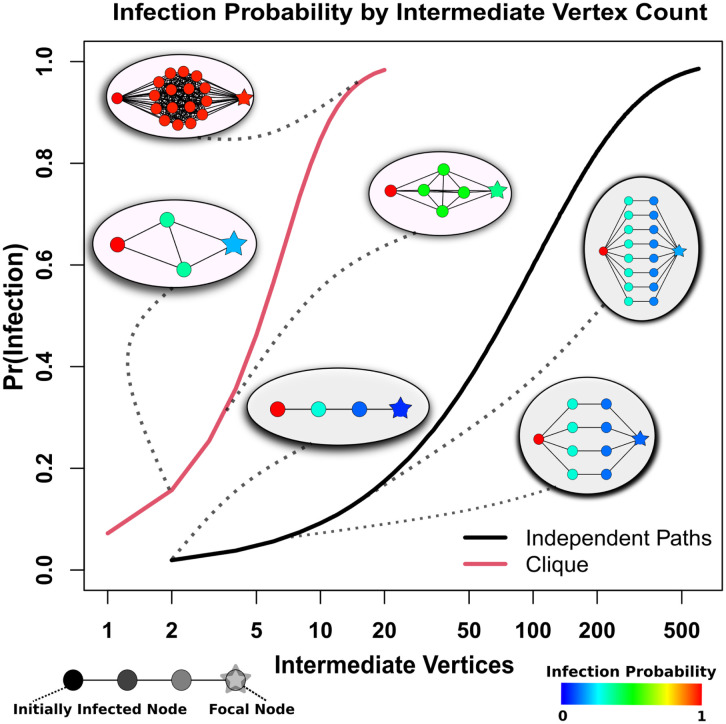
Probability of diffusion from an infected (*Left*) to uninfected (*Right*) individual bridged by intermediaries arranged in cliques (red curve) versus independent paths (black curve). Comembership in a cohesive subgroup fields infection risks that climb sharply with the number of intermediaries, while much larger numbers of intermediaries are required to obtain the same risk in the case of independent paths.

To determine whether these network effects would be expected to manifest under realistic conditions, we employ the above model (3) to study early pandemic infection hazards in the city of San Francisco, CA, a major city with a diverse population that suffered significant disparities in pandemic outcomes. We examine the period before March 24, 2020, 1 wk after infection data became available for the four major racial/ethnic groups; by this time, the infection was already spreading throughout the city, and significant racial and ethnic disparities in incidence had emerged. The observed patterns of disparity are typical of what would be expected given the underlying network process, with disparities in infection risks being greatly enhanced by differences in social cohesion. As we further show through simulation, these differences are expected to be geographically correlated, leading to a high-risk “floodplain” that is particularly exposed to infection, and metaphorical “high ground” that is relatively protected.

## Results

### Infection Outcomes.

**Fig. 2.**
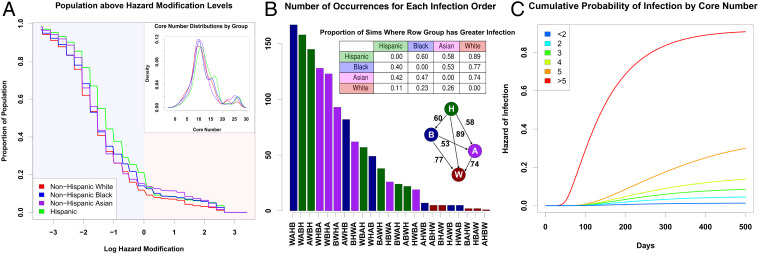
(*A*) Proportion of each population that lives “below” a given point on the floodplain (higher risk), denoted by its log hazard modification. The non-Hispanic White population is consistently present on the higher parts of the floodplain, with the non-Hispanic Asian population also being present in the middle of the floodplain. The lower parts of the floodplain are heavily occupied by non-Hispanic Black and Hispanic populations. (*Inset*) Distribution of core numbers for each ethnoracial group in the San Francisco model; small differences in core numbers are sufficient to drive large differences in risk. (*B*) Distribution of qualitative outcomes in simulation on March 24, where *x* axis labels correspond to group labels in order of infection rates, from lowest (bottom) to highest (top) prevalence. Bars are colored corresponding to the group with highest prevalence. The third bar (order AWBH) corresponds to the observed pattern from San Francisco. (*Top Inset*) The proportion of times each row group has a greater infection rate than the column group across all simulations. The Hispanic population consistently has the highest infection rates, followed, on average, by the Black population, the Asian population, and the non-Hispanic White population. (*Bottom Inset*) A graph describing the proportion of simulations one group (tail) has a greater infection rate than another (head). (*C*) Cumulative probability of infection by core number from simulated networks. Higher core numbers indicate greater levels of local cohesion, which substantially increases one’s hazard of infection. The bicomponent, where core number is equal to two, does not seem to drive infection patterns, as some prior literature suggests (8).

We simulate 1,225 infection trajectories (“pandemic histories”) for the city of San Francisco (*Materials and Methods*) covering the period up to March 24, 2020. Fig. 2*B* shows the resulting distribution of early infection disparities by demographic group (Hispanic [H], non-Hispanic Black [B], non-Hispanic White [W], and non-Hispanic Asian [A]) on March 24, 2020 of the simulation. Because outbreaks can vary greatly in size and timing, early period disparities can and do vary by trajectory. However, we see that Hispanics are hardest hit in the majority of cases, typically followed by Blacks and then Asians. Non-Hispanic Whites are very rarely the hardest hit, and are often (but not always) the group with the lowest early incidence; we note more variability in the identity of the least-hit group, as this outcome is sensitive to chance events (i.e., where early outbreaks occur). The observed pattern based on official data (9) is the third-most common pattern that would be expected, and hence fairly typical of what would be expected given the contact process.

### Cohesion Drives Infection Hazard.

Fig. 1 shows the risk-enhancing effect of cohesion in isolated subnetworks; this effect generalizes to more-realistic scenarios. A Cox proportional hazards model of infection hazard by core number (a common measure of embeddedness in cohesive groups) confirms a large risk enhancement for local cohesion, with persons in cohesive subgroups facing dramatically higher infection risk over time (Fig. 2*C*); in particular, each unit increment in core number increases infection hazard by ∼30%. Different demographic groups have slightly different levels of cohesion (Fig. 2 *A*, *Inset*). The difference in mean core number between the most cohesive group (Hispanic) and the least (non-Hispanic White) is 1.5, translating to an ∼50% mean risk enhancement; while risk levels vary within all groups, a 9.3% higher share of Hispanic versus non-Hispanic White population has greater than average risk (Fig. 2*A*). Differences in local social cohesion thus provide an important structural basis for disparities in early pandemic outcomes between groups.

### Spatial Correlation of Cohesion Produces a Network “Floodplain”.

**Fig. 3.**
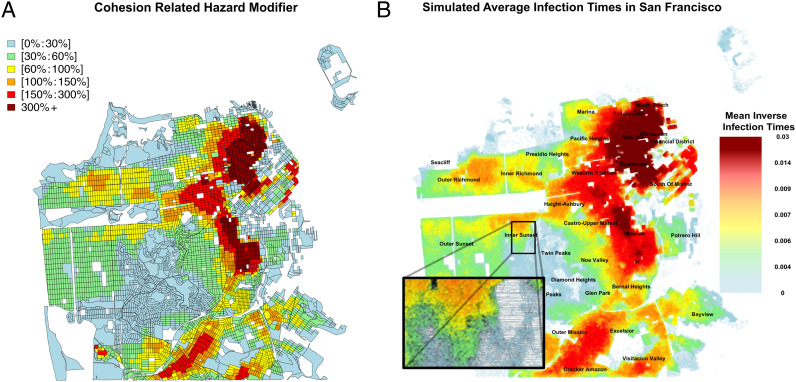
(*A*) Average deviation from the mean hazard attributable to core number, across San Francisco. Risk enhancement is spatially correlated, with significant risk downtown and much lower risk near the central part of the city. These hazards form a “floodplain,” where some areas are more dangerous than others. (*B*) Simulated infection times across San Francisco, averaged across 35 simulations. The patterns of infections match the expected hazard modifications in *A*. *Inset* shows the structure of the social network in the Inner Sunset neighborhood.

Contact network cohesion is spatially correlated, producing areas with higher than average membership in cohesive subgroups, and hence elevated mean risk. Fig. 3*A* shows the mean infection hazard modifier (net of global average) for each US Census block in San Francisco, based on the distribution of cohesion scores (core numbers). Cyan and green areas are epidemiological “high ground” where lower levels of local cohesion reduce mean risk, while red and orange areas are epidemiological “floodplains” where high cohesion leads to enhanced local risk. These cohesion-driven patterns are well correlated with the overall rate of infections, as illustrated by the mean inverse infection time across the city (Fig. 3*B*). Spatial segregation in housing places some groups in harm’s way, increasing disparities in incidence during the initial outbreak.

## Discussion

The mere presence of connecting paths is not sufficient for rapid diffusion of a disease like wild-type SARS-CoV-2: Infection of contacts is rare enough to require considerable redundancy for transmission to occur. Cohesion greatly increases the number of potential infection pathways, rendering an otherwise relatively “opaque” network “transparent” to disease transmission. The uneven distribution of cohesive subgroups in large networks and their much greater permeability help to explain the “bursty” nature of SARS-CoV-2 diffusion, with slow diffusion through less cohesive parts of the network punctuated by rapid outbreaks in cohesive groups (3, 10). Ironically, social cohesion has long been viewed as a community asset, particularly with respect to community resilience following disasters or other sources of social disruption (11–13); in the context of an infection like SARS-CoV-2, this same cohesion can act as an epidemiological risk factor. Local cohesion varies by location, with some parts of the San Francisco network having higher local cohesion than others. Combined with high levels of residential segregation, these differences can, in turn, produce disparities in infection hazard by race and ethnicity. In San Francisco, we find that Black and Hispanic populations are expected to have the highest infection rates in the early pandemic, followed by the Asian population and the White non-Hispanic population. Our models suggest that the exact evolution of infection rates is somewhat contingent on chance events, and multiple scenarios are possible based on which subgroups are hit first; however, some scenarios are much more likely than others, with the observed pattern of infection in the early pandemic being one of those predicted to be most likely to occur. Greater attention to cohesion as a risk factor—particularly given its spatial correlation—may help to prioritize warning messages or interventions for high-risk groups when outbreaks of a potentially serious disease are first detected.

## Materials and Methods

Population data for the COVID-19 simulation are from 2010 block-level US Census data for San Francisco. The number of observed infection cases of each racial group comes from San Francisco Department of Public Health (9). Contact network simulations and COVID-19 transmission employ the published model of ref. 3, with additional corrections for recovery and mortality hazards by age and sex as well as the date of the existence of patient 0, as described in *SI Appendix*. Model and parameterization details are contained in *SI Appendix*, along with the simulation details. Assessment of the cohesion/infection hazard relationship was performed via Cox proportional hazards models; parameterization details are provided in *SI Appendix*. Cross-tabulation of expected risk enhancement by areal unit and group produced the results of Figs. 2*A* and 3.

## Supplementary Material

Supplementary File

## Data Availability

R objects containing spatial Bernoulli networks and code for analysis of simulated network data have been deposited in Harvard Dataverse (https://doi.org/10.7910/DVN/NT4KDH) (14).

## References

[1] Centers for Disease Control and Prevention, CDC COVID data tracker. https://covid.cdc.gov/covid-data-tracker/#datatracker-home. Accessed 1 February 2021.

[2] T. Andrasfay, N. Goldman, Reductions in 2020 US life expectancy due to COVID-19 and the disproportionate impact on the Black and Latino populations. Proc. Natl. Acad. Sci. U.S.A. 118, e2014746118 (2021). 33446511 10.1073/pnas.2014746118 PMC7865122

[3] L. J. Thomas ., Spatial heterogeneity can lead to substantial local variations in COVID-19 timing and severity. Proc. Natl. Acad. Sci. U.S.A. 117, 24180–24187 (2020). 32913057 10.1073/pnas.2011656117 PMC7533653

[4] B. Hong, B. J. Bonczak, A. Gupta, L. E. Thorpe, C. E. Kontokosta, Exposure density and neighborhood disparities in COVID-19 infection risk. Proc. Natl. Acad. Sci. U.S.A. 118, e2021258118 (2021). 33727410 10.1073/pnas.2021258118 PMC8020638

[5] X. Hou ., Intracounty modeling of COVID-19 infection with human mobility: Assessing spatial heterogeneity with business traffic, age, and race. Proc. Natl. Acad. Sci. U.S.A. 118, e2020524118 (2021). 34049993 10.1073/pnas.2020524118 PMC8214685

[6] Centers for Disease Control and Prevention, COVID-19 hospitalization and death by race/ethnicity. https://www.cdc.gov/coronavirus/2019-ncov/covid-data/investigations-discovery/hospitalization-death-by-race-ethnicity.html. Accessed 1 February 2021.

[7] M. Morris, H. Epstein, M. Wawer, Timing is everything: International variations in historical sexual partnership concurrency and HIV prevalence. PLoS One 5, e14092 (2010). 21124829 10.1371/journal.pone.0014092 PMC2991312

[8] J. Moody, J. Adams, M. Morris, Epidemic potential by sexual activity distributions. Netw. Sci. (Camb. Univ. Press) 5, 461–475 (2017). 29449942 10.1017/nws.2017.3 PMC5809000

[9] San Francisco Department of Public Health, COVID-19 cases summarized by race and ethnicity. https://data.sfgov.org/COVID-19/COVID-19-Cases-Summarized-by-Race-and-Ethnicity/vqqm-nsqg. Accessed 21 April 2021.

[10] F. Wong, J. J. Collins, Evidence that coronavirus superspreading is fat-tailed. Proc. Natl. Acad. Sci. U.S.A. 117, 29416–29418 (2020). 33139561 10.1073/pnas.2018490117 PMC7703634

[11] C. Fan, Y. Jiang, A. Mostafavi, Emergent social cohesion for coping with community disruptions in disasters. J. R. Soc. Interface 17, 20190778 (2020). 32126194 10.1098/rsif.2019.0778 PMC7115229

[12] I. Townshend, O. Awosoga, J. Kulig, H. Fan, Social cohesion and resilience across communities that have experienced a disaster. Nat. Hazards 76, 913–938 (2015).

[13] J. E. Cinner ., Sixteen years of social and ecological dynamics reveal challenges and opportunities for adaptive management in sustaining the commons. Proc. Natl. Acad. Sci. U.S.A. 116, 26474–26483 (2019). 10.1073/pnas.1914812116 PMC6936519 31843883

[14] T. J. Loring ., Geographical Patterns of Social Cohesion Drive Disparities in Early COVID Infection Hazard. Harvard Dataverse. 10.7910/DVN/NT4KDH. Deposited 4 March 2022. PMC8944260 35286198

