# Supplementary material for: Geographical patterns of social cohesion drive disparities in early COVID infection hazard
Source: Proc Natl Acad Sci U S A. 2022 Mar 14;119(12):e2121675119. doi: 10.1073/pnas.2121675119 (PMC8944260; doi:10.1073/pnas.2121675119)
Supplement: Supplementary File [file pnas.2121675119.sapp.pdf]

PNAS
www.pnas.org

Supplementary Information for

Geographical Patterns of Social Cohesion Drive Disparities in Early COVID Infection Hazard

Loring J. Thomas, Peng Huang, Fan Yin, Junlan Xu, Zack W. Almquist, John R. Hipp, Carter T. Butts

Carter T. Butts.
E-mail: buttsc@uci.edu

This PDF file includes:

Supplementary text
SI References

Supporting Information Text

Introduction

In this appendix, we include additional information about the parameterization of the diffusion model, as well as the Cox Proportional-Hazards model. This section also provides more detail on the data that is used to generate the networks and estimate parameters.

Network and Demographic Data

We employ data from the 2010 U.S. Census to generate the population level social networks that underlie the analysis in this manuscript. Specifically, we use the smallest level of geography publicly available from the U.S. Census, known as the U.S. Census block level (approximately a city block in an urban setting). Each block contain basic demographic information, including household size.

To generate the network, we employ spatial network models that rely on a kernel function (the Spatial Interaction Functions, SIFs) to describe the presence of a social tie based on the distance between nodes; each node represents a single individual, and all simulations explicitly track the infection history of each individual in the population (as well as their infection paths). We employ the same network generation process used by Thomas et al. (1), which leverages the strategy of (2, 3) of placing households within Census blocks using a low-discrepancy (Halton) sequence, followed by jittered placement of individual locations about the household center. To parameterize the model used in this manuscript, we need to first define the spatial network models (or spatial Bernoulli models) which depend on the SIF. The SIF describes the probability of a tie being present between any two entities, given the distance between those entities. We use the same SIFs as in Thomas et al. (1) which employ a power law model of the form, $\mathcal{F}(\mathcal{D}_{ij}, \theta) = \frac{p_b}{(1+\alpha\mathcal{D}_{ij})^{\gamma}}$, where p_b describes the baseline probability of a tie existing, α is a scaling parameter describing the effect of a unit of distance, $\mathcal{D}_{ij}$ is the distance a dyad spans, and γ is a parameter describing the form of the tie probability decay. The simulation process employed uses two SIFs, based on prior literature to generate networks. The parameters for these SIFs can be found in (1).

Departing from prior work, we also leverage demographic information on U.S. Census blocks. These demographic covariates are race, ethnicity, age, and sex. These demographic covariates were assigned to nodes such that the three way distribution of race/sex/age and the two way distribution of race/ethnicity match the observed data at the block level. This allows a more fine-grained parameterization for simulation of the diffusion of COVID across social contact networks, based on demographic characteristics of each node (as detailed in the next section). We note that our procedure also leverages household size and thus represents the increased likelihood of being in a clique for individuals in such settings. This factor is one of the core factors that leads to COVID risk, as household spread of the disease is a primary avenue of spread.

We apply this technique to map social contact networks of San Francisco for three core reasons. (i) San Francisco is a city/county administrative unit – this is important because most data reported for the COVID-19 pandemic is at the county level in the U.S. and this allows us to analyze a complete city. (ii) San Francisco is a peninsula that is separated on three sides by water, reducing boundary effects from contacts outside the border of the city. (iii) The city/county of San Francisco published longitudinal data on infections by ethno-racial groups of the early pandemic (4). The combination of good data management and reporting makes San Francisco unique, and when taken together with its status as a natural reporting unit (i.e. also being a county) it becomes an important unique case for studies such as the one conducted in this manuscript. We observe that future decisions by other municipalities to publish longitudinal data broken down by demographics would facilitate further studies of this kind.

In general the epidemiological literature has shown that population density increases the rate of disease spread (5, 6), but it does not provide a mechanistic interpretation for this phenomenon. However, previous research on spatial network models has highlighted the way in which density can drive tie creation and resulting cohesive subgroup formation (3). Our model provides a specific mechanism for how population density and household size distributions may result in increased disease spread: population distribution influences the creation of locally cohesive regions within the contact network, and these regions are *exceptionally permeable* to SARS-CoV-2. It is important to observe that this is not equivalent to number of contacts *per se* - as shown in Fig. 1, susceptibles with large numbers of contacts may still have relatively low infection hazard, when not embedded in a highly cohesive group.

Parameterization of Diffusion Model

To simulate the spread of COVID across a social contact network, we use a continuous time diffusion model defined by (1). This diffusion model describes the way that individuals in the social network experience the disease and spread it to others. This diffusion model begins with the network structure and a vector of disease states for each node (individual). Disease states can be Susceptible (an individual who does not have the disease, but can get infected with it), Infected (the individual has been infected with the disease, but is not infectious), Infectious (the individual can spread the disease to others), Dead, or Recovered. At the beginning of the simulation, all nodes begin in the Susceptible state, with the exception of the seed infections. These nodes begin the simulation being infected with the disease. 25 individuals, randomly selected from the population, are the seed infections in each of the simulations.

Simulations are run until a steady state has been achieved, in which there are no more infected or infectious people, with everyone being in the Susceptible, Recovered, or Dead states. At this point, the diffusion model provides a detailed history

for each node, describing the individual's final state in the simulation, as well as the times at which the node entered any given state. The disease spreads across the structure of the network, with connected nodes being able to transmit the disease across their social ties. Infection occurs as a Poisson event with a fixed rate, described by (1). Only infectious nodes can infect susceptible social contacts; once an individual recovers or dies, they are no longer able to infect or be infected with COVID. When a Susceptible node is infected by an infectious alter, a Bernoulli trial is performed, determining whether a node becomes terminally or non-terminally infected. The rate of success (terminal infection) of the Bernoulli trial is given by P_d, a matrix sorted by age in the row and sex in the column (top to bottom row: 0-9, 10-19, 20-29, 30-39, 40-49, 50-59, 60-69, 70-79, and 80+; left to right column: female and male); for an individual with age category i and sex category j, the indicator for terminal infection thus arises as $T_{ij} \sim \mathrm{Bern}(P_{dij})$. P_d, which is in essence a transformation of the Infection Fatality Ratio (IFR) broken down by age and sex, is calculated based on two pieces of information: the IFR for each age group (7), and the sex ratio of death probability within each age group (8), assuming the probability of male and female getting infected is equal within each age group. P_d describes the set of Bernoulli parameters determining the likelihood of a fatal infection:

$$P_d = \begin{pmatrix} 0.000022 & 0.000018 \\ 0.000049 & 0.000049 \\ 0.000216 & 0.000384 \\ 0.000604 & 0.000996 \\ 0.001045 & 0.001955 \\ 0.003625 & 0.008375 \\ 0.012360 & 0.036410 \\ 0.030357 & 0.071643 \\ 0.070189 & 0.115811 \end{pmatrix}$$

The timing of transitions between different states is governed by a series of Gamma distributions. The waiting time from being infected to being infectious is governed by a Gamma distribution with shape 5.807 and scale 0.948, as estimated by (9). For transition towards recovery or death, while prior work used homogeneous distributions, we break them down by demographics to more accurately account for variation across different populations. We estimate their parameters by matching the mean and standard deviation of waiting time for each group, using epidemiological data reported in (10–12). These method of moments estimators coincide with maximum likelihood estimators for the associated parameters, given that the Gamma distribution is a member of the exponential family. Specifically, the waiting time to death for a terminally infected individual in age category i is distributed as ${t^d}_i \sim \mathrm{Gamma}(G_{di1}, G_{di2})$, where G_d is a parameter matrix whose columns contain shape and rate parameters, respectively, and rows indicate age category (top to bottom: 0-49, 50-64, and 65+). (Note that we do not vary the waiting time distribution by sex, as we are not aware of applicable time-to-mortality data from the early pandemic that supports age/sex decomposition.) Here, G_d is given as follows:

$$G_d = \begin{pmatrix} 3.744 & 0.251 \\ 3.568 & 0.233 \\ 2.881 & 0.223 \end{pmatrix}$$

The waiting time to recovery is broken down by both age and sex. For a male in age category i with a non-terminal infection, the waiting time to recovery is distributed as $t^r_{im} \sim \mathrm{Gamma}(G^{rm}_{i1}, G^{rm}_{i2})$, where G^{rm} is a parameter matrix whose rows are ordered by age category (top to bottom: 0-19, 20-29, 30-39, 40-49, 50-59, 60+) and whose columns respectively contain shape and rate parameters. Here, G^{rm} is given as follows:

$$G^{rm} = \begin{pmatrix} 5.339 & 0.392 \\ 5.782 & 0.414 \\ 5.808 & 0.402 \\ 6.686 & 0.452 \\ 6.301 & 0.425 \\ 6.242 & 0.424 \end{pmatrix}$$

For a non-terminally infected female in age category i, the waiting time to recovery is similarly distributed as $t^r_{if} \sim \mathrm{Gamma}(G^{rf}_{i1}, G^{rf}_{i2})$, where G^{rf} is a second parameter matrix whose rows are also ordered by age category (top to bottom: 0-19, 20-29, 30-39, 40-49, 50-59, 60+) and whose columns respectively contain shape and rate parameters. G^{rf} is as follows:

$$G^{rf} = \begin{pmatrix} 5.395 & 0.408 \\ 5.623 & 0.402 \\ 5.326 & 0.376 \\ 6.258 & 0.424 \\ 5.776 & 0.407 \\ 4.719 & 0.337 \end{pmatrix}$$

Since the diffusion process precedes the reporting of the first confirmed positive case, we performed a grid search to determine the length of the time lag between the appearance of "patient zero" in the city and the report of the first positive confirmed

case (March 3, 2021 (13)). Our search was performed over an interval from a minimum of 1 and a maximum of 100 days. For each possible number, we regressed the number of infection case for each racial group in their observed time period using data from (4), on its counterparts in the simulation. The loss function is the summation of the mean squared errors (MSE) for all the linear regressions. We find that a 35 day lag minimizes the MSE, and this value is used here.

Simulation Details

Given the network and diffusion models described above, we run a series of simulations in which the population of San Francisco is seeded with randomly placed infectives 35 days prior to the first confirmed case report in San Francisco on March 3, 2021, and the infection process is followed until the end of our observation period (March 24, 2020, one week after demographic data becomes available for all four major racial/ethnic groups within the city). 35 individual-level contact networks were generated for San Francisco, using different simulated node locations for each realization. For each of these 35 simulated networks, we run 35 diffusion replicates, reseeding the seed infections for each simulation. This produces 1225 simulation replicates.These networks were produced with the R programming language, using the `sna` library (14, 15). For results reported about a single network realization in the main text, we average the infection time (or inverse infection time) for each diffusion replicate simulated in that network. The network being averaged across was selected as the network that most closely matches the average infection and susceptibility splits across all networks on March 24, 2020. For other metrics (such as the reported Cox model results), we average across the entire sample of networks. All figures from the main text utilize simulated data calibrated to observed data on infections and deaths.

The number of replications (independently simulated networks and diffusion simulations within network) was chosen based on a preliminary power analysis based on pilot simulations. Due to the diffusion simulation being bound to the structure of the social network, multiple network replicates were used to highlight trends in infection patterns across space. Likewise, given that the pandemic trajectories are dependent on the seed locations in the network, we randomized the seeds in each pandemic replicate to ensured that simulated trends were not due to idiosyncrasies in seed placement in the network structure. (The equality between the replication count and the inferred optimal lag time for the first infection is coincidental.)

Cox Proportional-Hazards Models

To assess the effects of local cohesion on infection hazards, we use Cox Proportional-Hazards models. Cox models control for (possibly time-varying) background hazards, allowing us to identify the impact of cohesion on infection hazard net of the overall progress of the outbreak. Because each simulated outbreak follows a distinct trajectory, we fit a single model to each simulated trajectory (with the baseline hazard, plus a single effect for core number). This model predicts the hazard of an uninfected individual getting infected with COVID-19, using the core number of a given node (16) as a cohesion measure. The *core number* of a node - specifically, the highest k such that the node belongs to the kth degree core of the contact network - is a measure of local cohesion, with higher numbers indicating that the focal node is embedded in a more cohesive subgroup. In particular, nodes with core numbers of 0 are isolates, those of core 1 belong to trees or pendant trees, and those of core number 2 or higher belong to bicomponents (with higher numbers indicating higher levels of of cohesion). The core number is measured in units of ties, with a core number of k indicating that ego has at least k ties to alters who themselves have core numbers of at least k (and hence who have at least k ties to others with at least k ties to others in the core, recursively). We note that core number is not equivalent to degree: one can have arbitrarily high degree and still have a core number as low as 1. The Cox model coefficient for core number thus indicates the extent to which nodes embedded in locally cohesive regions within the contact network are infected more or less rapidly (on average) than other nodes, controlling for the time-varying baseline infection hazard.

The form of the Cox used here is $h(t) = h_b(t)\exp(\beta X)$. Here, $h(t)$ represents the infection hazard, with $h_b(t)$ being the baseline hazard, X the core number, and β a coefficient expressing the increase in the log infection hazard per unit increase in core number. Here, we observed a mean β of 0.2615 over all simulations, implying an average risk enhancement of approximately 30% in infection hazard per unit increase in core number (as reflected in Fig.2C). As described in the main text, cohesion is a strong and consistent risk factor for early COVID infection, with nodes in high-order cores having a much higher infection risk than those in low-order cores.

Code and Data Availability

We have provided the code and data used for this project, including all parameters for the demographic models. This archive can be found at https://doi.org/10.7910/DVN/NT4KDH.

References

1. Thomas LJ, et al. (2020) Spatial Heterogeneity can Lead to Substantial Local Variations in COVID-19 Timing and Severity. *Proceedings of the National Academy of Sciences* 117(39):24180–24187.
2. Almquist ZW, Butts CT (2012) Point Process Models for Household Distributions Within Small Areal Units. *Demographic Research* 26:593–632.
3. Butts CT, Acton RM, Hipp JR, Nagle NN (2012) Geographical variability and network structure. *Social Networks* 34:82–100.

4. San Francisco Department of Public Health (2021) COVID-19 Cases Summarized by Race and Ethnicity (https://data.sfgov.org/COVID-19/COVID-19-Cases-Summarized-by-Race-and-Ethnicity/vqqm-nsqg). Accessed: 4/21/2021.
5. Kadi N, Khelfaoui M (2020) Population density, a factor in the spread of COVID-19 in Algeria: Statistical study. *Bulletin of the National Research Centre* 44(1):1–7.
6. Rashed EA, Kodera S, Gomez-Tames J, Hirata A (2020) Influence of absolute humidity, temperature and population density on COVID-19 spread and decay durations: Multi-prefecture study in Japan. *International Journal of Environmental Research and Public Health* 17(15):5354.
7. Ferguson, Neil and Laydon, Daniel and Nedjati Gilani, Gemma and Imai, Natsuko and Ainslie, Kylie and Baguelin, Marc and Bhatia, Sangeeta and Boonyasiri, Adhiratha and Cucunuba Perez, ZULMA and Cuomo-Dannenburg, Gina and others (2020) Impact of non-pharmaceutical interventions (NPIs) to reduce COVID19 mortality and healthcare demand (https://www.imperial.ac.uk/media/imperial-college/medicine/sph/ide/gida-fellowships/Imperial-College-COVID19-NPI-modelling-16-03-2020.pdf). Accessed: 11/18/2021.
8. Bhopal SS, Bhopal R (2020) Sex Differential in COVID-19 Mortality Varies Markedly by Age. *Lancet (London, England)*.
9. Lauer, Stephen A and Grantz, Kyra H and Bi, Qifang and Jones, Forrest K and Zheng, Qulu and Meredith, Hannah R and Azman, Andrew S and Reich, Nicholas G and Lessler, Justin (2020) The incubation period of coronavirus disease 2019 (COVID-19) from publicly reported confirmed cases: Estimation and application. *Annals of Internal Medicine* 172(9):577–582.
10. Voinsky I, Baristaite G, Gurwitz D (2020) Effects of Age and Sex on Recovery from COVID-19: Analysis of 5769 Israeli Patients. *Journal of Infection* 81(2):e102–e103.
11. Khalili M, et al. (2020) Epidemiological Characteristics of COVID-19: a Systematic Review and Meta-analysis. *Epidemiology & Infection* 148.
12. CDC (2020) CDC COVID-19 Pandemic Planning Scenarios (https://cdc.gov/coronavirus/2019-ncov/hcp/planning-scenarios.html). Accessed: 9/7/2020.
13. San Francisco Department of Public Health (2021) COVID-19 Cases Over Time (https://data.sfgov.org/COVID-19/COVID-19-Cases-Over-Time/gyr2-k29z). Accessed: 10/07/2021.
14. Butts CT (2008) Social Network Analysis with sna. *Journal of Statistical Software* 24(6):1–51.
15. R Core Team (2013) R: A Language and Environment for Statistical Computing.
16. Seidman SB (1983) Network structure and minimum degree. *Social Networks* 5:269–287.
